# Exposure to Negative News Stories About Vaping, and Harm Perceptions of Vaping, Among Youth in England, Canada, and the United States Before and After the Outbreak of E-cigarette or Vaping-Associated Lung Injury (‘EVALI’)

**DOI:** 10.1093/ntr/ntac088

**Published:** 2022-04-03

**Authors:** Katherine East, Jessica L Reid, Robin Burkhalter, Olivia A Wackowski, James F Thrasher, Harry Tattan-Birch, Christian Boudreau, Maansi Bansal-Travers, Alex C Liber, Ann McNeill, David Hammond

**Affiliations:** School of Public Health Sciences, Faculty of Health, University of Waterloo, Waterloo, ON, Canada; National Addiction Centre, Institute of Psychiatry, Psychology and Neuroscience, King’s College London, London, UK; School of Public Health Sciences, Faculty of Health, University of Waterloo, Waterloo, ON, Canada; School of Public Health Sciences, Faculty of Health, University of Waterloo, Waterloo, ON, Canada; School of Public Health, Rutgers, The State University of New Jersey, Piscataway, NJ, USA; Arnold School of Public Health, University of South Carolina, Columbia, SC, USA; Department of Behavioural Science and Health, Institute of Epidemiology and Health Care, University College London, London, UK; Department of Statistics and Actuarial Science, University of Waterloo, ON, Canada; Department of Health Behavior, Roswell Park Comprehensive Cancer Center, Buffalo, NY, USA; Cancer Prevention and Control, Lombardi Comprehensive Cancer Center, Georgetown University, Washington, DC, USA; National Addiction Centre, Institute of Psychiatry, Psychology and Neuroscience, King’s College London, London, UK; School of Public Health Sciences, Faculty of Health, University of Waterloo, Waterloo, ON, Canada

## Abstract

**Introduction:**

Little is known about the international impact of E-cigarette or Vaping-Associated Lung Injury (‘EVALI’) on youth perceptions of vaping harms.

**Methods:**

Repeat cross-sectional online surveys of youth aged 16–19 years in England, Canada, and the United States before (2017, 2018), during (2019 August/September), and after (2020 February/March, 2020 August) the ‘EVALI’ outbreak (N = 63380). Logistic regressions assessed trends, country differences, and associations between exposure to negative news stories about vaping and vaping harm perceptions.

**Results:**

Exposure to negative news stories increased between 2017 and February–March 2020 in England (12.6% to 34.2%), Canada (16.7% to 56.9%), and the United States (18.0% to 64.6%), accelerating during (2019) and immediately after (February–March 2020) the outbreak (*p* < .001) before returning to 2019 levels by August 2020. Similarly, the accurate perception that vaping is less harmful than smoking declined between 2017 and February–March 2020 in England (77.3% to 62.2%), Canada (66.3% to 43.3%), and the United States (61.3% to 34.0%), again accelerating during and immediately after the outbreak (*p* < .001). The perception that vaping takes less than a year to harm users’ health and worry that vaping will damage health also doubled over this period (*p* ≤ .001). Time trends were most pronounced in the United States. Exposure to negative news stories predicted the perception that vaping takes less than a year to harm health (Adjusted Odds Ratio = 1.55, 1.48-1.61) and worry that vaping will damage health (Adjusted Odds Ratio = 1.32, 1.18-1.48).

**Conclusions:**

Between 2017 and February–March 2020, youth exposure to negative news stories, and perceptions of vaping harms, increased, and increases were exacerbated during and immediately after ‘EVALI’. Effects were seen in all countries but were most pronounced in the United States.

**Implications:**

This is the first study examining changes in exposure to news stories about vaping, and perceptions of vaping harms, among youth in England, Canada, and the United States before, during, and after ‘EVALI’. Between 2017 and February–March 2020, youth exposure to negative news stories, and perceptions of vaping harms, increased, and increases were exacerbated during and immediately after ‘EVALI’. By August 2020, exposure to negative news stories returned to 2019 levels, while perceptions of harm were sustained. Exposure to negative news stories also predicted two of the three harm perception measures. Overall, findings suggest that ‘EVALI’ may have exacerbated youth’s perceptions of vaping harms internationally.

## Introduction

Beginning March 2019, there was an outbreak of serious lung injury in the United States dubbed ‘EVALI’ (E-cigarette or Vaping-Associated Lung Injury).^1^ The number of hospital admissions from ‘EVALI’ peaked in September 2019, and by February 2020 the US Centers for Disease Control and Prevention (CDC) reported 2 807 hospitalized cases and 68 deaths.^1^ Many lines of evidence now indicate that ‘EVALI’ was primarily caused by vaping liquids containing vitamin E acetate—an additive in some illicit cannabis vaping products but not used in nicotine e-cigarettes.^1–4^ The outbreak was largely localized to the United States in geographically concentrated clusters.^2^ The CDC reported that, among 2 022 patients who were hospitalized from ‘EVALI’ with data on substance use, 82% self-reported vaping tetrahydrocannabinol (THC; ie, cannabis) and 14% self-reported exclusively vaping nicotine e-cigarettes.^1^ Canada also reported 20 cases^5^; of which, 40% self-reported vaping THC. The United Kingdom reported two possible cases, which were both fatal and associated with the recent use of vaping synthetic cannabis.^6,7^

The outbreak was widely reported in the media.^8–11^ In the United States, 62% of all news articles about e-cigarettes published in 2019 mentioned ‘EVALI’,^9^ and news reporting peaked in September 2019.^8–10^ The articles that mentioned the outbreak were often accompanied by warnings of the health harms of vaping, concerns about youth vaping, and were less likely to mention that vaping is less risky than smoking.^9,10^ There was also a greater emphasis on avoiding the use of all vaping products than avoiding vaping THC specifically,^10^ and on deaths related to vaping rather than vaping contaminated THC.^9^ Outside of the US, preliminary data indicate that the prevalence of news articles about e-cigarettes also peaked in September 2019 in Canada and the United Kingdom.^11^ However, there has been little research examining the extent to which the public report noticing negative news stories about vaping around this time, in the United States or internationally.

Research suggests that ‘EVALI’ has had a widespread impact on vaping perceptions and related behaviors. There is a strong consensus that, although not risk-free, vaping is less harmful than smoking.^12–14^ However, in the United States, the outbreak was associated with increased perceptions of the health harms of nicotine vaping among youth^15,16^ and adults,^15,17–19^ including misperceptions that vaping is more harmful than smoking.^17,18^ These perceptions were sustained even after vitamin E acetate-contaminated cannabis vaping was implicated as the primary cause,^17^ and knowledge of this as the main cause of ‘EVALI’ remained low even in 2021, over a year after the outbreak.^20^ The outbreak was also associated with an increase in internet searches for vaping cessation^8^ and a decrease in e-cigarette sales^15^ and online vape shop searches^21^ in the United States. Among adults in England, inaccurately perceiving that vaping is more harmful than smoking also increased after the outbreak, suggesting an international impact.^22,23^ To the best of our knowledge, there has been no research in Canada comparing harm perceptions of vaping before versus after ‘EVALI’.

More broadly, surveys and experimental studies have found that information about vaping in the media can change vaping harm perceptions. Exposure to e-cigarette advertisements has been associated with reductions in the perceived harmfulness of vaping among youth and adults,^24,25^ while exposure to anti-vaping campaigns and news headlines can increase the perceived harmfulness of vaping among US adults.^25–27^ Harm perceptions of vaping relative to smoking have also been associated with the portrayal of vaping in the media among adult smokers,^28^ while providing accurate information about vaping can correct vaping misperceptions^29^ and inaccurate information can increase vaping misperceptions.^30^

Monitoring vaping perceptions among youth is important because misperceptions are pervasive among this age group,^31^ are often resistant to correction,^32^ and could be maintained into adulthood. Despite this, there has been little research examining how ‘EVALI’ has impacted perceptions of vaping harm among youth outside of the United States. There has also been little research directly comparing changes in harm perceptions in countries that were differentially impacted by ‘EVALI’. Monitoring inaccurate perceptions of vaping is particularly important because they could act as a barrier to vaping among smokers.^33,34^

This study of youth in England, Canada, and the United States, therefore, aimed to examine: (1) changes over time and between countries in self-reported exposure to negative news stories about vaping, and perceptions of vaping harms and (2) associations between exposure to negative news stories about vaping and perceptions of vaping harms. Specific hypotheses were:

1. Compared with prior to the outbreak (2017 and 2018), exposure to negative news stories about vaping would be more commonly reported during (August–September 2019), while perceptions of vaping harms would be greater during (August–September 2019) and after (February–March and August 2020), the outbreak.
2. Changes in exposure to negative news stories about vaping, and perceptions of vaping harms, would be greater in the United States (which had the greatest number of ‘EVALI’ cases and deaths) than Canada (which had 20 documented cases), with England having the smallest changes.
3. Exposure to negative news stories about vaping would be positively associated with perceptions of vaping harms.

## Methods

The analysis plan was pre-registered, and code made available, on the Open Science Framework (osf.io/buqh8).^35^

### Data source

Data were from the International Tobacco Control Policy Evaluation Project (ITC) Youth Tobacco and Vaping Survey, a repeat cross-sectional survey conducted in England, Canada, and the United States. A full description of the study methods can be found in the technical reports.^36–38^ Briefly, online surveys were conducted in 2017 (24 July to 29 August), 2018 (2 August to 24 September), 2019 (14 August to 14 September), February–March 2020 (6 February to 2 March), and August 2020 (7 to 31 August). Respondents aged 16–19 years were recruited through the Nielsen Consumer Insights Global Panel and received remuneration according to their panel’s incentive structure. This study received ethics clearance through the University of Waterloo Research Ethics Committee (ORE#21847/31017) and the King’s College London Psychiatry, Nursing & Midwifery Research Ethics Subcommittee.

A total of 70 063 respondents completed the surveys; of whom, 63 380 were retained in this study’s analytic sample. Respondents were excluded if they failed data integrity checks (*n* = 2 290), had missing/incomplete data on variables required for calculating weights or determining smoking or vaping status (*n* =1 783), were recruited in a previous wave (*n* = 2 220), were an ineligible age (*n* = 106), and, for this study only, selected “Refused” on the outcome variables (*n* = 284). Sample characteristics are shown in Supplementary Table S1).

### Measures

#### Outcomes

##### Exposure to Mostly Negative News Stories About Vaping.

 “In the last 30 days, about how often, if at all, have you seen or heard a NEWS story about e-cigarettes/vaping?” Respondents who answered “Rarely,” “Sometimes,” “Often,” or “Very often” were then asked “Were the majority of news stories you saw or heard about e-cigarettes…” with responses coded as mostly negative (“Mostly negative about e-cigarettes”) versus other (“Mostly positive about e-cigarettes,” “About the same number of positive and negative stories,” “Don’t know”). Respondents who answered “Never” or “Don’t know” to the frequency of noticing item were also coded as “other”.

##### Accurate Perception That Vaping is Less Harmful Than Smoking.

“Is using e-cigarettes/vaping less harmful, about the same, or more harmful than smoking cigarettes?” with responses coded as less harmful (“A lot less harmful than ‘regular’ tobacco cigarettes,” “A little less harmful than ‘regular’ tobacco cigarettes”) versus other (“As harmful as ‘regular’ tobacco cigarettes,” “A little more harmful than ‘regular’ tobacco cigarettes,” “A lot more harmful than ‘regular’ tobacco cigarettes”, “Don’t know”).

##### Perception That Vaping Takes Less Than a Year to Harm Users’ Health.

“How long do you think someone has to use e-cigarettes/vape before it harms their health?” with responses coded as “less than a year” versus other (“It will never harm their health,” “1 year,” “5 years,” “10 years,” “20 years or more,” “Don’t know”). This dichotomization was selected to reflect the acute onset of ‘EVALI’.

##### Worry That Vaping Will Damage Your Health in the Future (Among Past 30-day Vapers).

 Past 30-day vapers-only were asked, “Are you worried that using e-cigarettes/vaping will damage your health in the future?” with responses coded as very/moderately worried (“Very worried,” “Moderately worried”) versus other (“A little worried,” “Not at all worried,” “Don’t know”).

The full distributions of responses for the above outcomes by country and survey wave are shown in Supplementary Tables S2–S4.

#### Independent Variables

*Country.* England, Canada, and the United States.
*Survey wave*. 2017, 2018, 2019, February–March 2020, August 2020; treated as categorical to aid interpretation of the findings.

#### Covariates

*Age g*roup. 16–17 years, 18–19 years.
*Sex*. Male, female.
*Race/*ethnicity. White-only, any other race/ethnicity, don’t know/refused.
*Vaping and smoking status (Aim 2 only)*. When examining associations between exposure to negative news stories about vaping and perceptions of vaping harms, vaping status (never, ever but not past 30-day, past 30-day) and smoking status (never, ever but not past 30-day, past 30-day) were included as covariates.

### Analysis

Analyses were conducted using Stata, v.16.

First, descriptive statistics for study outcomes were reported by country and survey wave. To address Aim 1, adjusted (for demographic covariates) logistic regression models were used to predict each outcome from survey wave and country. A country-by-survey wave interaction term was subsequently added to the adjusted logistic regression models, and interactions were examined further via contrasts within countries using Stata’s *margins* command. The following were also run: (1) separate subgroup analyses by vaping and smoking status groups and (2) sensitivity analyses including an indicator of survey month in 2019 (August versus September), because news reporting of the outbreak varied during the data collection period, peaking in September 2019.^8–11^

To get further insight on how outcomes evolved over time, in an additional step that was not pre-registered,^35^ the change between Jully–August 2017 and August–September 2018 (ie, prior to ‘EVALI’) was compared with the change between August–September 2018 and February–March 2020 (ie, prior to, during, and immediately after ‘EVALI’). To this end, Stata’s *lincom* command was used to test the contrast comparing those two slopes. Because the time period for the first trend (1.0833 years on average) was shorter than the time period for the second trend (1.5 years on average), a sensitivity analysis compared the relative change by dividing the change between July–August 2017 and August–September 2018 by 1.0833, and the change between August–September 2018 and February–March 2020 by 1.5. The findings remained unchanged.

To address Aim 2, adjusted (for all above covariates, country, and survey wave) logistic regression models were used to predict each of the three vaping harm perception measures (entered uniquely into the model) from exposure to negative news stories. In an additional step that was not pre-registered,^35^ a harm perception-by-country interaction term was subsequently added to the adjusted logistic regression models, and interactions were examined further via contrasts within countries using Stata’s *margins* command.

Cross-sectional post-stratification sample weights were applied in all analyses. See Technical Reports for details.^36–38^ Briefly, weights were constructed for each country, calibrated to sex-by-age-by-region in Canada and England and sex-by-age-by-region-by-race in the United States; student status; school grades; and past 30-day smoking trend in Canada and the United States, and rescaled to each country’s sample size.

## Results

### Aim 1. Examine Changes Over Time and Across Countries in Exposure to Negative News Stories About Vaping and Perceptions of Vaping Harms

**Figure 1.**
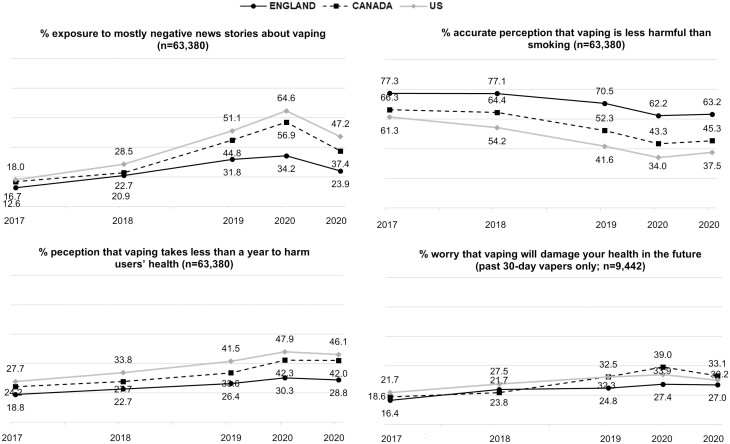
Trends in Exposure to Mostly Negative News Stories About Vaping and Perceptions of Vaping Harms Between 2017 and 2020 in England, Canada, and the United States. Data are Weighted and Unadjusted.

Figure 1 shows the trends in exposure to mostly negative news stories about vaping and perceptions of vaping harms over time in each country. Tables 1 and 2 show the findings from the adjusted regression models aggregated across countries, and Table 3 shows the contrasts between survey waves within each country.

**Table 1.** Associations Between Exposure to Mostly Negative News Stories and Perceptions of Vaping Harms and survey wave (2017–2020) and Country, Adjusting for Demographic Covariates

	Full sample (*n* = 63 380)									
	*n*	Exposure to mostly negative news stories about vaping			Accurate perception that vaping is less harmful than smoking			Perception that vaping takes less than a year to harm users' health		
		%	AOR (95% CI)	*p*	%	AOR (95% CI)	*p*	%	AOR (95% CI)	*p*
Survey wave										
2017 (Jul–Aug)	12067	15.8	REF		68.2	REF		23.6	REF	
2018 (Aug–Sep)	11713	24.1	1.74 (1.62-1.87)	<.001	65.1	0.88 (0.83-0.94)	<.001	28.1	1.29 (1.20-1.37)	<.001
2019 (Aug–Sep)	11549	43.1	4.14 (3.85-4.44)	<.001	54.1	0.55 (0.51-0.58)	<.001	34.1	1.68 (1.58-1.80)	<.001
2020 (Feb–Mar)	13564	52.7	6.16 (5.75-6.59)	<.001	45.8	0.39 (0.36-0.41)	<.001	40.6	2.22 (2.09-2.37)	<.001
2020 (Aug)	14487	37.5	3.17 (2.96-3.39)	<.001	47.4	0.42 (0.40-0.45)	<.001	39.8	2.12 (2.00-2.26)	<.001
Country										
England	19851	24.7	REF		69.8	REF		25.5	REF	
Canada	20396	36.1	1.85 (1.76-1.95)	<.001	54.0	0.51 (0.49-0.54)	<.001	34.2	1.63 (1.55-1.71)	<.001
United States	23133	43.3	2.41 (2.29-2.54)	<.001	44.6	0.35 (0.33-0.36)	<.001	40.3	1.97 (1.87-2.07)	<.001
Survey wave * country interaction^a^										
		*F*(8,63372) = 24.49, *p* < .001			*F*(8,63372) = 6.17, *p* < .001			*F*(8,63372) = 3.83, *p* < .001		

All data except sample *n* are weighted. The full regression models including associations with demographic covariates are shown in Supplementary Table S6. Associations using August–September 2019 and Canada as the reference categories are shown in Supplementary Table S7.

Interactions were added in a second step to the regression models.

**Table 2.** Associations Between Exposure to Mostly Negative News Stories and worry that vaping will damage your health in the future and survey wave (2017–2020) and Country, Adjusting for Demographic Covariates

	Past 30-day vapers (*n* = 9442)			
	Worry that vaping will damage your health in the future			
	*n*	%	AOR (95% CI)	*p*
Survey wave				
2017 (Jul–Aug)	1090	19.2	REF	
2018 (Aug–Sep)	1493	24.7	1.34 (1.08-1.65)	.008
2019 (Aug–Sep)	2220	30.6	1.81 (1.48-2.20)	<.001
2020 (Feb–Mar)	2751	34.0	2.14 (1.77-2.59)	<.001
2020 (Aug)	1888	30.2	1.81 (1.48-2.21)	<.001
Country				
England	2244	24.5	REF	
Canada	3298	31.1	1.30 (1.13-1.49)	<.001
United States	3900	30.1	1.31 (1.14-1.51)	<.001
Survey wave * country interaction^a^		*F*(8,9434) = 2.01, *p* = .042		

All data except sample *n* are weighted. The full regression models including associations with demographic covariates are shown in Supplementary Table S6. Associations using Aug–Sep 2019 and Canada as the reference categories are shown in Supplementary Table S7.

Interactions were added in a second step to the regression models.

**Table 3.** Contrasts Between Survey Waves (2017–2020) Within Each Country for Exposure to Mostly Negative News Stories and Perceptions of Vaping Harms, Adjusting for Demographic Covariates.

	Full sample (*n* = 63 380)						Past 30-day vapers (*n* = 9 442)	
	Exposure to mostly negative news stories about vaping		Accurate perception that vaping is less harmful than smoking		Perception that vaping takes less than a year to harm users’ health		Worry that vaping will damage your health in the future	
	AOR (95% CI)	*p*	AOR (95% CI)	*p*	AOR (95% CI)	*p*	AOR (95% CI)	*P*
England								
2017 (Jul–Aug)	REF		REF		REF		REF	
2018 (Aug–Sep)	1.09 (1.07–1.11)	<.001	1.00 (0.98–1.02)	.929	1.04 (1.02–1.06)	<.001	1.08 (1.01–1.15)	.032
2019 (Aug–Sep)	1.21 (1.19–1.24)	<.001	0.93 (0.91–0.96)	<.001	1.08 (1.06–1.10)	<.001	1.09 (1.02–1.15)	.007
2020 (Feb–Mar)	1.24 (1.22–1.26)	<.001	0.86 (0.84–0.88)	<.001	1.12 (1.10–1.14)	<.001	1.12 (1.05–1.18)	<.001
2020 (Aug)	1.12 (1.10–1.14)	<.001	0.87 (0.85–0.89)	<.001	1.11 (1.08–1.13)	<.001	1.11 (1.05–1.18)	.001
Canada								
2017 (Jul–Aug)	REF		REF		REF		REF	
2018 (Aug–Sep)	1.07 (1.05–1.09)	<.001	0.99 (0.97–1.02)	.593	1.04 (1.02–1.07)	<.001	1.02 (0.96–1.08)	.575
2019 (Aug–Sep)	1.33 (1.30–1.36)	<.001	0.87 (0.85–0.89)	<.001	1.10 (1.08–1.13)	<.001	1.14 (1.07–1.21)	<.001
2020 (Feb–Mar)	1.50 (1.47–1.53)	<.001	0.80 (0.78–0.82)	<.001	1.20 (1.18–1.23)	<.001	1.22 (1.15–1.30)	<.001
2020 (Aug)	1.23 (1.21–1.26)	<.001	0.81 (0.79–0.83)	<.001	1.20 (1.17–1.23)	<.001	1.15 (1.08–1.23)	<.001
United States								
2017 (Jul–Aug)	REF		REF		REF		REF	
2018 (Aug–Sep)	1.11 (1.09–1.13)	<.001	0.93 (0.91–0.95)	<.001	1.06 (1.04–1.09)	<.001	1.06 (1.00–1.12)	.043
2019 (Aug–Sep)	1.39 (1.36–1.42)	<.001	0.82 (0.80–0.84)	<.001	1.15 (1.12–1.18)	<.001	1.11 (1.05–1.18)	<.001
2020 (Feb–Mar)	1.59 (1.56–1.63)	<.001	0.76 (0.74–0.78)	<.001	1.22 (1.19–1.25)	<.001	1.13 (1.07–1.20)	<.001
2020 (Aug)	1.34 (1.31–1.37)	<.001	0.79 (0.77–0.81)	<.001	1.20 (1.18–1.23)	<.001	1.09 (1.03–1.16)	.003

Estimates were obtained using Stata’s margins command following a survey wave*country interaction term added to the adjusted logistic regression models shown in Tables 1 and 2.

Exposure to mostly negative news stories about vaping increased between 2017 and February–March 2020 overall (Table 1) and within each of England (12.6% to 34.2%), Canada (16.7% to 56.9%), and the United States (18.0% to 64.6%) (Table 3). As hypothesized, compared with prior to the outbreak, exposure to negative news stories was greater during the outbreak (2019) and, when contrasting the slopes of the increase from 2017 to 2018 with the increase from 2018 to February–March 2020, there was strong evidence for the contrast (*p* < .001), indicating that the increase accelerated during and immediately after the outbreak. By August 2020, exposure to negative news stories returned to below 2019 levels (Adjusted Odds Ratio [AOR] = 0.77, 95% CI = 0.72–0.81; Supplementary Table S7).

Perceptions of vaping harms followed a similar pattern, such that accurate perceptions that vaping is less harmful than smoking decreased between 2017 and February/March 2020 overall (Table 1) and within England (77.3% to 62.2%), Canada (66.3% to 43.3%), and the United States (61.3% to 34.0%) (Table 3), while perceptions that vaping takes less than a year to harm users’ health and past 30-day vapers’ worry that vaping will damage their health almost doubled over this period (Tables 1–3). As hypothesized, compared with prior to the outbreak, perceptions of vaping harms were greater during (2019) and after (February–March and August 2020) the outbreak. There was also strong evidence that the trends in perceptions of vaping harms (but not worry among past 30-day vapers; *p* = .275) accelerated during and immediately after the outbreak, when contrasting the increase from 2017 to 2018 with the increase from 2018 to February–March 2020 (*p* < .001). Unlike exposure to negative news stories, in August 2020 all perceptions of vaping harms remained greater than or equal to 2019 levels (Supplementary Table S7).

Overall, exposure to mostly negative news stories about vaping and perceptions of vaping harms were greater in Canada and the United States than in England (Table 1 and 2), and, except for past 30-day vapers’ worry that vaping will damage health in the future, greater in the United States than in Canada (Supplementary Table S7). There was also evidence for an interaction between survey wave and country for all four outcomes (Table 1 and 2). As hypothesized, between 2017 and February/March 2020, exposure to negative news stories increased to a greater extent in the United States (AOR = 2.30, 1.94–2.72, *p* < .001) and Canada (AOR = 1.84, 1.55–2.17, *p* < .001) than in England, and to a greater extent in the United States than in Canada (AOR = 1.25, 1.06–1.40, *p* = .008) (data not shown in tables). Similarly, the accurate perception that vaping is less harmful than smoking also decreased over this same period to a greater extent in the United States (AOR = 0.67, 0.58–0.77, *p* < .001) and Canada (AOR = 0.80, 0.69-0.93, *p* = .004) than in England, and to a greater extent in the United States than in Canada (AOR = 0.83, 0.72–0.96, *p* = .011). The perception that vaping takes less than a year to harm users’ health increased to a greater extent in the United States (AOR = 1.28, 1.10–1.49, *p* = .002) and Canada (AOR = 1.24, 1.06–1.44, *p* = .007) than England, but trends were similar in Canada vs. the United States (AOR = 1.04, 0.89–1.20, *p* = .648). There was little evidence that trends in past 30-day vapers’ worry that vaping will damage their health in the future varied between the United States and Canada (AOR = 0.69, 0.43–1.09, *p* = .110), England and the United States (AOR = 0.98, 0.61–1.56, *p* = .919), or England and Canada (AOR = 1.42, 0.86–2.37, *p* = .173) between 2017 and February–March 2020 (data not shown in tables).

#### Subgroup Analysis

All trends and country differences were broadly similar among never-, ever-, and past 30-day smokers, and never-, ever-, and past 30-day vapers (Supplementary Tables S8–S13). However, some interactions between country and survey wave were no longer statistically significant for some groups (eg, past 30-day smokers, Supplementary Table S10), possibly due to lower sample sizes.

#### Sensitivity Analysis

Trends were similar when 2019 data were separated into August versus September data collection months (Supplementary Table S14). When directly comparing these months, exposure to mostly negative news stories about vaping was greater in September 2019 —during the peak of the outbreak – than in August 2019, while the three harm perceptions measures were similar (Supplementary Table S14).

### Aim 2. Associations Between Exposure to Negative News Stories About Vaping and Perceptions of Vaping Harms

**Figure 2.**
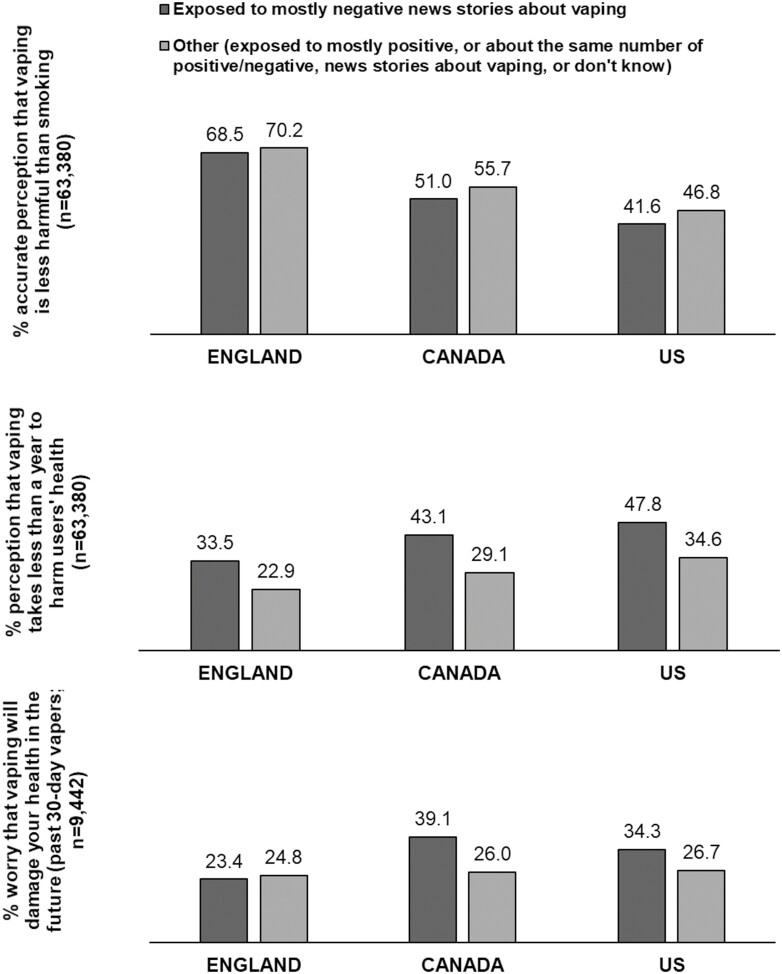
Associations Between Exposure to Mostly Negative News Stories About Vaping and Perceptions of Vaping Harms in Each of England, Canada, and the United States (2017– 2020). Data are Weighted and Unadjusted.

Figure 2 shows the associations between exposure to mostly negative news stories about vaping and perceptions of vaping harms in each country. As hypothesized, overall there was strong evidence that participants reporting exposure to mostly negative news stories about vaping (vs. otherwise) had greater odds of perceiving that vaping takes less than a year to harm users’ health (43.1% vs. 28.6%; AOR = 1.55, 1.48–1.61, *p* < .001) and, among past 30-day vapers, being worried that vaping will damage their health in the future (34.2% vs. 25.9%; AOR = 1.32, 1.18–1.48, *p* < .001) (data not shown in tables). Contrary to hypothesized, exposure to mostly negative news stories about vaping showed little overall association with the accurate perception that vaping is less harmful than smoking after adjusting for covariates (50.6% vs. 58.1%; AOR = 1.00, 0.96–1.04, *p* = .947).

Examining interactions, there was little evidence that associations between exposure to mostly negative news stories and accurate perception that vaping is less harmful than smoking (*F*_(df=2,63378)_ = 0.52, *p* = .595) and perception that vaping takes less than a year to harm users’ health (*F*_(df=2,63378)_ = 1.68, *p* = .187) differed by country. However, there was an interaction between exposure to mostly negative news stories and country when predicting past 30-day vapers’ worry that vaping will damage their health (*F*_(df=2,9440)_ = 10.10, *p* < .001), such that the association was stronger in Canada (AOR = 2.01, 1.48–2.73, *p* < .001) and the United States (AOR = 1.59, 1.17–2.17, *p* = .003) than in England. Examining these interactions further within countries indicated that exposure to mostly negative news stories was associated with worry that vaping will damage users’ health only among past 30-day vapers from Canada (AOR = 1.11, 1.08–1.16, *p* < .001) and the United States (AOR = 1.06, 1.02–1.10, *p* = .002), but not England (AOR = 0.97, 0.93–1.01, *p* = .147).

## Discussion

To the best of our knowledge, this is the first study to examine changes in exposure to news stories about vaping and perceptions of vaping harms among youth within and outside of the United States before and after ‘EVALI’. All three hypotheses were generally supported. First, youth exposure to negative news stories and perceptions of vaping harms increased over the study period, with the greatest increases observed during and immediately after the outbreak. Consistent with prior research from the United States,^15–19^ and among adults in England,^22,23^ perceptions of harms from vaping were greater during the outbreak and sustained through to August 2020. Exposure to negative news stories was also greatest in the period immediately after the outbreak. Second, effects were generally strongest in the United States, which had the greatest number of ‘EVALI’ cases and deaths,^1,5,6^ followed by Canada, which had 20 documented cases.^5^ Third, consistent with prior research,^25,27,28^ youth who were exposed to negative news stories about vaping also perceived greater harms from vaping across two measures: The perception that vaping takes less than a year to harm users’ health and, among past 30-day vapers, worry that vaping will damage their health in the future. Taken together, findings suggest ‘EVALI’ may have exacerbated perceptions of vaping harms among youth internationally.

Contrary to hypothesized, and inconsistent with the trends observed and findings for the other two harm perception measures, there was little evidence for an association between exposure to negative news stories and accurately perceiving vaping as less harmful than smoking. This is also inconsistent with surveys among adult smokers and ex-smokers which have found cross-sectional associations between perceived media portrayal of vaping and perceived relative harmfulness of vaping,^28^ although youth are a distinct group from adult smokers/ex-smokers. The differences in associations between the three harm perceptions measures and exposure to negative news stories in this study may relate to measure specificity. That is, the perception that vaping takes less than a year to harm users’ health and the worry that vaping will damage health may have shown associations with negative news stories because ‘EVALI’ demonstrated acute and severe effects; however, perceptions of vaping *relative* to smoking among youth may be less directly impacted by news stories. Further research is required to replicate and further examine this finding among youth.

Youth from the United States consistently perceived the greatest harm from vaping, followed by Canada, then England. In addition to ‘EVALI’, country differences may be partially attributable to e-cigarette regulations,^39^ public health messaging,^29^ youth vaping prevention campaigns (particularly in the United States),^40^ social norms,^31^ and general media portrayal vaping,^28,41^ of which have all been associated with vaping harm perceptions. Within countries, the general increases seen in perceptions of vaping harms over time are consistent with national trends in England^12^ and the United States.^42^ To the best of our knowledge, no comparable national trends are available in Canada, although a survey among adult vapers in Canada found that perceptions of vaping harms increased between 2019 and 2020.^43^

Subgroup analyses found that time trends were similar when split by smoking and vaping status, suggesting that perceptions of the harms from vaping have increased since 2017 and were exacerbated during/after ‘EVALI’, regardless of whether youth smoked or vaped. The increase in vaping harm perceptions among past 30-day smokers is particularly concerning because this group has the most to gain from understanding the lower relative risk of vaping, and hence should be targeted by interventions to correct misperceptions.

Findings may help to understand how ‘EVALI’ has impacted vaping perceptions and may help to guide how vaping is communicated by the media in the future. While previous data have shown that ‘EVALI’ media coverage peaked in September 2019,^8-11^ this study was the first to demonstrate that youth *noticed* more negative vaping news stories around this time, and that perceptions of vaping harms also increased and were generally associated with noticing news stories. These findings are consistent with prior studies finding that media can shape vaping harm perceptions.^25–30,44^ Media reporting should therefore distinguish between the mode of administration (eg, vaping, smoking) and what is being consumed (eg, nicotine, illicit products). Media coverage and public education campaigns aiming to correct misperceptions of nicotine vaping, including misperceptions of what actually caused ‘EVALI’,^20^ may also be helpful.

Research is needed to understand the extent to which the observed trends in perceptions translate to vaping and smoking behaviors. At the individual level, increases in perceptions of harms from vaping could act as a barrier to smokers using e-cigarettes to help them to quit or reduce smoking.^33,34^ However, at the population-level, even as perceptions of vaping harms have increased,^12,42,43^ vaping prevalence has also generally increased,^12,45–50^ particularly among youth in Canada and the United States,^45,48,49^ despite the slight decrease among United States youth immediately after ‘EVALI’ but before the Coronavirus Disease 2019 (COVID-19) pandemic.^51^ This discrepancy between trends in population-level perceptions of vaping harms and vaping prevalence among youth may be because youth report a range of reasons for using e-cigarettes, including curiosity, for fun, popularity among friends, or for the flavors, as well as perceptions of reduced harm relative to smoking.^12,52,53^ Continued monitoring of vaping perceptions, alongside vaping and smoking behaviors, among youth and adults is important to help further understand their association.

This study is not without limitations. First, the reduction in exposure to negative news stories and perceptions of vaping harms in August 2020 may be partially attributable to COVID-19 impacting vaping behaviors^54,55^ and dominating news coverage at that time. However, COVID-19 could not explain the substantial increases in news exposure and harm perceptions of vaping observed in 2019 and February/March 2020, which are the primary focus of this study. Second, the measures used in this study did not pertain to ‘EVALI’ specifically (eg, exposure to news stories about ‘EVALI’) and the harm perception measures did not distinguish between vaping nicotine and vaping contaminated cannabis products (the primary cause of ‘EVALI’). Misreporting of e-cigarette use with cannabis vaping was negligible in the ITC Youth surveys in 2018^56^; regardless, harm perceptions of nicotine versus cannabis vaping warrants future research. Third, the measures do not specify news story content, which may have been broader than ‘EVALI’. In 2019, 62% of US news articles about e-cigarettes mentioned ‘EVALI’,^9^ although there are no comparable estimates in Canada and England. Fourth, survey weights differed between countries: data for Canada and the United States were weighted to reflect national smoking trends among youth, while data for England were not due to a lack of national smoking estimates among English youth aged 16–19 years. However, prevalence estimates in the ITC Youth survey are similar to national benchmark surveys,^36–38^ and the large effect sizes observed in this study are unlikely to be an effect of survey weighting.

Explanations aside from ‘EVALI’ may also exist for the increases observed in exposure to negative news stories and perceptions of vaping harms. E-cigarette policies and policy recommendations have changed between 2017 and 2020; for example, flavor bans and nicotine limits came into force in several Canadian provinces.^57^ Bans on some flavored e-cigarette products were also announced in the United States around the same time as ‘EVALI’. Reporting of ‘EVALI’ is also often conflated with reporting of concerns about youth vaping.^9^ It is, therefore, difficult to disentangle the effects of ‘EVALI’ from news stories about increasing restrictions and youth vaping. Despite this, our finding that trends in exposure to negative news stories accelerated during (August–September 2019) and immediately after (February–March 2020) the ‘EVALI’ outbreak, combined with the several studies showing that ‘EVALI’-specific media coverage peaked in September 2019,^8–11^ suggests some specificity of our findings to ‘EVALI’.

Strengths of this study include the use of data from three countries that were differentially impacted by ‘EVALI’, the convergence of key findings across three measures of vaping harm perceptions, and the large sample that allowed for subgroup analyses by smoking and vaping status.

## Conclusions

Between 2017 and February–March 2020, exposure to negative news stories and harm perceptions of vaping increased among youth in England, Canada, and the United States, and trends were exacerbated during and immediately after the 2019 ‘EVALI’ outbreak. Effects were observed in all three countries but were strongest in the United States, which had most ‘EVALI’ cases. Findings highlight a need to better distinguish between, and communicate the risks of, vaping nicotine e-liquids and vaping contaminated illicit products.

## Supplementary Material

A Contributorship Form detailing each author’s specific involvement with this content, as well as any supplementary data, are available online at https://academic.oup.com/ntr.

ntac088_suppl_Supplementary_Tables

ntac088_suppl_Supplementary_Taxonomy-form

## Data Availability

This manuscript describes analyses of secondary data. The code is available online at osf.io/buqh8.^35^
