# Supplementary material for: Exposure to Negative News Stories About Vaping, and Harm Perceptions of Vaping, Among Youth in England, Canada, and the United States Before and After the Outbreak of E-cigarette or Vaping-Associated Lung Injury (‘EVALI’)
Source: Nicotine Tob Res. 2022 Apr 3;24(9):1386–95. doi: 10.1093/ntr/ntac088 (PMC9356695; doi:10.1093/ntr/ntac088)
Supplement: ntac088_suppl_Supplementary_Tables [file ntac088_suppl_supplementary_tables.docx]

**Table S1. Sample characteristics (n=63,380) Data are weighted % (unweighted n).**

|  | **ENGLAND** | | | | | | | | | |  | | **CANADA** | | | | | | | | | | |  | | **US** | | | | | | | | |
| --- | --- | --- | --- | --- | --- | --- | --- | --- | --- | --- | --- | --- | --- | --- | --- | --- | --- | --- | --- | --- | --- | --- | --- | --- | --- | --- | --- | --- | --- | --- | --- | --- | --- | --- |
|  | **2017**  **(Jul-Aug)** | | **2018**  **(Aug-Sep)** | | **2019**  **(Aug-Sep)** | | **2020**  **(Feb-Mar)** | | **2020**  **(Aug)** | |  | | **2017**  **(Jul-Aug)** | | **2018**  **(Aug-Sep)** | | **2019**  **(Aug-Sep)** | | **2020**  **(Feb-Mar)** | | **2020**  **(Aug)** | | |  | **2017**  **(Jul-Aug)** | | | **2018**  **(Aug-Sep)** | | **2019**  **(Aug-Sep)** | | **2020**  **(Feb-Mar)** | | **2020**  **(Aug)** |
|  | **n=3971** | | **n=3867** | | **n=3484** | | **n=4255** | | **n=4274** | |  | | **n=4015** | | **n=3820** | | **n=4109** | | **n=4198** | | **n=4254** | | |  | **n=4081** | | | **n=4026** | | **n=3956** | | **n=5111** | | **n=5959** |
| **AGE GROUP^1^** |  | |  | |  | |  | |  | |  | |  | |  | |  | |  | |  | | |  |  | | |  | |  | |  | |  |
| 16-17 | 48.6  (1596) | | 48.4  (1168) | | 48.5  (1513) | | 48.9  (1871) | | 49.0  (1660) | |  | | 47.8  (1573) | | 47.3  (1452) | | 47.2  (1770) | | 47.2  (1717) | | 47.8  (2180) | | |  | 50.1  (1790) | | | 49.4  (2040) | | 49.4  (1676) | | 49.1  (2430) | | 49.2  (3032) |
| 18-19 | 51.4  (2375) | | 51.6  (2699) | | 51.5  (1971) | | 51.1  (2384) | | 51.0  (2614) | |  | | 52.2  (2442) | | 52.7  (2368) | | 52.8  (2339) | | 52.8  (2481) | | 52.2  (2074) | | |  | 49.9  (2291) | | | 50.6  (1986) | | 50.6  (2280) | | 50.9  (2681) | | 50.8  (2927) |
| **SEX^1^** |  | |  | |  | |  | |  | |  | |  | |  | |  | |  | |  | | |  |  | | |  | |  | |  | |  |
| Male | 51.2  (1685) | | 51.3  (1395) | | 51.2  (1228) | | 51.4  (1603) | | 51.4  (1558) | |  | | 51.3  (1399) | | 51.4  (1770) | | 51.3  (1538) | | 51.3  (1604) | | 51.2  (1706) | | |  | 51.0  (1608) | | | 51.0  (1436) | | 51.1  (1218) | | 50.9  (1690) | | 51.0  (1745) |
| Female | 48.8  (2286) | | 48.7  (2472) | | 48.8  (2256) | | 48.6  (2652) | | 48.6  (2716) | |  | | 48.7  (2616) | | 48.6  (2050) | | 48.7  (2571) | | 48.7  (2594) | | 48.8  (2548) | | |  | 49  (2473) | | | 49  (2590) | | 48.9  (2738) | | 49.1  (3421) | | 49.0  (4214) |
| **RACE/ETHNICTY^1^** | |  | |  | |  | |  | |  | |  | |  | |  | |  | |  | |  |  | | | |  | |  | |  | |  | |
| White (only) | 79.6  (3118) | | 77.1  (2890) | | 76.0  (2616) | | 77.3  (3268) | | 74.9  (3124) | |  | | 58.5  (2164) | | 47.2  (1809) | | 53.9  (2234) | | 55.4  (2330) | | 56.3  (2424) | | |  | 73.4  (2673) | | | 73.3  (2483) | | 73.7  (2161) | | 73.3  (3105) | | 70.3  (2934) |
| Any other race/ethnicity | 19.4  (815) | | 21.7  (932) | | 22.7  (816) | | 21.3  (926) | | 24.1  (1102) | |  | | 39.7  (1776) | | 45.6  (1719) | | 43.3  (1754) | | 42.0  (1755) | | 41.5  (1732) | | |  | 26.2  (1385) | | | 25.9  (1509) | | 25.8  (1760) | | 26.3  (1976) | | 29.2  (2960) |
| Don’t know/refused | 1.0  (38) | | 1.2  (45) | | 1.3  (52) | | 1.4  (61) | | 0.9  (48) | |  | | 1.9  (75) | | 7.2  (292) | | 2.8  (121) | | 2.6  (113) | | 2.1  (98) | | |  | 0.5  (23) | | | 0.7  (34) | | 0.5  (35) | | 0.4  (30) | | 0.5  (65) |
| **SMOKING STATUS^1^** | |  | |  | |  | |  | |  | |  | |  | |  | |  | |  | |  |  | | | |  | |  | |  | |  | |
| Never smoker | 59.6  (2326) | | 59.9  (2273) | | 61.8  (2093) | | 54.5  (2179) | | 60.2  (2474) | |  | | 68.1  (2811) | | 69.1  (2411) | | 69.0  (2618) | | 65.5  (2481) | | 72.6  (3051) | | |  | 67.7  (2745) | | | 67.6  (2757) | | 66.4  (2424) | | 67.9  (3270) | | 73.2  (4375) |
| Ever (but not past 30-day) smoker | 24.9  (1008) | | 23.4  (956) | | 23.3  (803) | | 25.9  (1137) | | 24.6  (1115) | |  | | 21.2  (815) | | 21.1  (830) | | 21.7  (937) | | 25.5  (1101) | | 18.7  (840) | | |  | 21.3  (894) | | | 20.7  (821) | | 25.6  (986) | | 25.1  (1207) | | 20.6  (1093) |
| Past 30-day smoker | 15.5  (637) | | 16.6  (638) | | 14.8  (588) | | 19.6  (939) | | 15.2  (685) | |  | | 10.7  (389) | | 9.8  (579) | | 9.2  (554) | | 9.0  (616) | | 8.7  (363) | | |  | 11.0  (442) | | | 11.6  (448) | | 7.9  (546) | | 7.0  (634) | | 6.2  (491) |
| **VAPING STATUS** | |  | |  | |  | |  | |  | |  | |  | |  | |  | |  | |  |  | | | |  | |  | |  | |  | |
| Never vaper | 66.4  (2639) | | 66.9  (2578) | | 63.9  (2141) | | 58.3  (2331) | | 62.8  (2565) | |  | | 70.7  (2885) | | 66.8  (2383) | | 59.5  (2207) | | 54.8  (2007) | | 64.1  (2677) | | |  | 68.7  (2752) | | | 67.0  (2677) | | 56.5  (2066) | | 57.7  (2705) | | 63.7  (3736) |
| Ever (but not past 30-day) vaper | 25.1  (997) | | 24.1  (975) | | 23.6  (871) | | 27.7  (1283) | | 26.2  (1227) | |  | | 21.0  (832) | | 21.2  (878) | | 22.8  (1024) | | 26.2  (1183) | | 23.2  (1022) | | |  | 20.1  (872) | | | 17.4  (729) | | 25.0  (1020) | | 23.5  (1304) | | 22.8  (1372) |
| Past 30-day vaper | 8.6  (335) | | 9.0  (314) | | 12.5  (472) | | 14.0  (641) | | 11.0  (482) | |  | | 8.3  (298) | | 12.0  (559) | | 17.7  (878) | | 19.0  (1008) | | 12.7  (555) | | |  | 11.1  (457) | | | 15.7  (620) | | 18.5  (870) | | 18.8  (1102) | | 13.6  (851) |

^1^ Note that age group, sex, race/ethnicity (US only), and past 30-day smoking (Canada and US only) were included in the survey weighting procedures.

**Table S2. Participant responses to ‘Were the majority of news stories you saw or heard about e-cigarettes…’ by country and survey wave (N=63,380). Data are weighted % (unweighted n).**

|  | **2017**  **(Jul-Aug)** | **2018**  **(Aug-Sep)** | **2019**  **(Aug-Sep)** | **2020**  **(Feb-Mar)** | **2020**  **(Aug)** |
| --- | --- | --- | --- | --- | --- |
| **ENGLAND** |  |  |  |  |  |
| Mostly negative | 26.6 (493) | 35.9 (862) | 49.7 (1102) | 53.2 (1431) | 45.7 (1050) |
| Mostly positive | 18.3 (352) | 20.3 (427) | 17.1 (388) | 14.7 (435) | 15.6 (375) |
| About the same number of positive and negative | 33.7 (625) | 34.1 (746) | 25.3 (571) | 24 (665) | 27.3 (619) |
| Don’t know | 21.4 (404) | 9.7 (218) | 7.9 (186) | 8.1 (225) | 11.3 (264) |
|  |  |  |  |  |  |
| **CANADA** |  |  |  |  |  |
| Mostly negative | 34.7 (661) | 41.7 (828) | 63.3 (1823) | 74.4 (2399) | 65.8 (1596) |
| Mostly positive | 13.9 (268) | 14.5 (334) | 10.3 (297) | 7.1 (235) | 7.3 (166) |
| About the same number of positive and negative | 30.1 (566) | 31 (664) | 19.6 (601) | 14.1 (462) | 19.1 (460) |
| Don’t know | 21.4 (435) | 12.9 (265) | 6.8 (193) | 4.4 (137) | 7.9 (200) |
|  |  |  |  |  |  |
| **US** |  |  |  |  |  |
| Mostly negative | 39.9 (728) | 51.1 (1163) | 68.4 (1941) | 80.4 (3269) | 73.7 (2771) |
| Mostly positive | 14.1 (273) | 13.8 (318) | 8.6 (302) | 5.3 (242) | 7.8 (322) |
| About the same number of positive and negative | 31.1 (568) | 25.9 (581) | 18 (556) | 10.8 (472) | 13.6 (581) |
| Don’t know | 15 (283) | 9.2 (214) | 4.9 (156) | 3.5 (152) | 4.8 (223) |

**Table S3. Participant responses to ‘Is using e-cigarettes/vaping less harmful, about the same, or more harmful than smoking cigarettes?’ by country and survey wave (N=63,380). Data are weighted % (unweighted n).**

|  | **2017**  **(Jul-Aug)** | **2018**  **(Aug-Sep)** | **2019**  **(Aug-Sep)** | **2020**  **(Feb-Mar)** | **2020**  **(Aug)** |
| --- | --- | --- | --- | --- | --- |
| **ENGLAND** |  |  |  |  |  |
| A lot more harmful | 1.3 (51) | 1.9 (66) | 2.5 (99) | 4.0 (189) | 2.8 (136) |
| A little more harmful | 2.0 (82) | 2.8 (105) | 4.2 (155) | 6.0 (273) | 5.9 (269) |
| As harmful | 10.1 (391) | 10.8 (399) | 14.9 (541) | 19.7 (862) | 17.9 (792) |
| A little less harmful | 37.4 (1526) | 41.8 (1742) | 44.5 (1526) | 40.7 (1691) | 40.5 (1740) |
| A lot less harmful | 40.0 (1561) | 35.3 (1303) | 26 (870) | 21.5 (877) | 22.7 (909) |
| Don't know | 9.3 (360) | 7.3 (252) | 8 (293) | 8.1 (363) | 10.2 (428) |
|  |  |  |  |  |  |
| **CANADA** |  |  |  |  |  |
| A lot more harmful | 1.8 (66) | 2.9 (114) | 5.5 (243) | 8.8 (371) | 6 (261) |
| A little more harmful | 2.8 (105) | 4.1 (175) | 6.4 (271) | 10.1 (447) | 8.2 (363) |
| As harmful | 16 (601) | 17.5 (657) | 23.3 (957) | 27.9 (1121) | 29.3 (1245) |
| A little less harmful | 39.5 (1662) | 38.2 (1443) | 34.9 (1415) | 31.6 (1306) | 34.3 (1444) |
| A lot less harmful | 26.8 (1083) | 26.2 (1006) | 17.4 (712) | 11.8 (519) | 11.0 (462) |
| Don't know | 13.2 (498) | 11.1 (425) | 12.4 (511) | 9.8 (434) | 11.1 (479) |
|  |  |  |  |  |  |
| **US** |  |  |  |  |  |
| A lot more harmful | 2.2 (88) | 3.2 (135) | 8.8 (368) | 12.3 (657) | 9 (616) |
| A little more harmful | 3.5 (145) | 5.4 (213) | 9 (373) | 11.1 (587) | 10.3 (639) |
| As harmful | 19.9 (770) | 26 (1042) | 30.2 (1177) | 32.7 (1641) | 33.6 (1971) |
| A little less harmful | 39.1 (1628) | 35.8 (1492) | 27.9 (1070) | 22.8 (1134) | 28.1 (1653) |
| A lot less harmful | 22.2 (921) | 18.4 (687) | 13.7 (512) | 11.3 (567) | 9.4 (505) |
| Don't know | 13.1 (529) | 11.2 (457) | 10.3 (456) | 9.9 (525) | 9.6 (575) |

**Table S4. Participant responses to ‘How long do you think someone has to use e-cigarettes/vape before it harms their health?’ by country and survey wave (N=63,380). Data are weighted % (unweighted n).**

|  | **2017**  **(Jul-Aug)** | **2018**  **(Aug-Sep)** | **2019**  **(Aug-Sep)** | **2020**  **(Feb-Mar)** | **2020**  **(Aug)** |
| --- | --- | --- | --- | --- | --- |
| **ENGLAND** |  |  |  |  |  |
| It will never harm their health | 6.3 (239) | 5.3 (183) | 3.7 (134) | 2.7 (125) | 2.9 (119) |
| Less than a year | 18.8 (760) | 22.7 (914) | 26.4 (900) | 30.3 (1294) | 28.8 (1215) |
| 1 year | 16.5 (666) | 19.3 (791) | 23.6 (826) | 22.7 (953) | 22.1 (976) |
| 5 years | 14 (556) | 14.7 (577) | 15.1 (553) | 15.9 (673) | 15.8 (709) |
| 10 years | 5.6 (226) | 5.5 (207) | 5.9 (207) | 5.9 (255) | 5.9 (250) |
| 20 years or more | 4.1 (160) | 3.8 (136) | 3.9 (131) | 4.1 (180) | 3.8 (165) |
| Don't know | 34.6 (1364) | 28.8 (1059) | 21.5 (733) | 18.5 (775) | 20.7 (840) |
|  |  |  |  |  |  |
| **CANADA** |  |  |  |  |  |
| It will never harm their health | 5.1 (196) | 4.2 (174) | 2.6 (112) | 2 (78) | 1.1 (45) |
| Less than a year | 24.2 (974) | 27.7 (1039) | 33.6 (1386) | 42.3 (1727) | 42 (1801) |
| 1 year | 16.2 (677) | 19.6 (754) | 24 (985) | 22.9 (995) | 22.2 (935) |
| 5 years | 15.1 (607) | 14.2 (538) | 14.5 (591) | 13.5 (580) | 12.5 (536) |
| 10 years | 5.3 (202) | 7.2 (258) | 5.2 (205) | 4.2 (187) | 4.9 (200) |
| 20 years or more | 3.5 (137) | 4.5 (178) | 3.9 (165) | 3.1 (130) | 2.7 (112) |
| Don't know | 30.6 (1222) | 22.6 (879) | 16.1 (665) | 12 (501) | 14.6 (625) |
|  |  |  |  |  |  |
| **US** |  |  |  |  |  |
| It will never harm their health | 4.7 (182) | 3.5 (140) | 2 (99) | 1.8 (108) | 1.2 (73) |
| Less than a year | 27.7 (1121) | 33.8 (1330) | 41.5 (1535) | 47.9 (2355) | 46.1 (2660) |
| 1 year | 16.5 (693) | 17.9 (747) | 20.2 (797) | 18.8 (1016) | 21.8 (1321) |
| 5 years | 14.7 (595) | 13.2 (547) | 13.1 (552) | 11.5 (605) | 11.7 (736) |
| 10 years | 5.6 (223) | 5.3 (200) | 4.9 (221) | 4.2 (212) | 4.5 (302) |
| 20 years or more | 3.9 (154) | 3.7 (156) | 3.6 (156) | 2.9 (148) | 3.1 (209) |
| Don't know | 26.9 (1113) | 22.6 (906) | 14.7 (596) | 12.9 (667) | 11.6 (658) |

**Table S5. Participant responses to ‘Are you worried that using e-cigarettes/vaping will damage your health in the future?’ by country and survey wave, among past 30-day vapers (N=9,442). Data are weighted % (unweighted n).**

|  | **2017**  **(Jul-Aug)** | **2018**  **(Aug-Sep)** | **2019**  **(Aug-Sep)** | **2020**  **(Feb-Mar)** | **2020**  **(Aug)** |
| --- | --- | --- | --- | --- | --- |
| **ENGLAND** |  |  |  |  |  |
| Not at all worried | 42.4 (146) | 40.5 (123) | 34.4 (155) | 28.4 (179) | 28.2 (140) |
| A little worried | 32.4 (104) | 31.2 (106) | 36.3 (170) | 40.2 (259) | 37.4 (183) |
| Moderately worried | 13.4 (45) | 20.8 (61) | 19.3 (97) | 21.9 (141) | 21.3 (102) |
| Very worried | 3 (10) | 2.9 (9) | 5.4 (27) | 5.5 (38) | 5.7 (29) |
| Don't know | 8.8 (30) | 4.6 (15) | 4.5 (23) | 4 (24) | 7.5 (28) |
|  |  |  |  |  |  |
| **CANADA** |  |  |  |  |  |
| Not at all worried | 39.5 (123) | 35.2 (198) | 21.8 (200) | 19.5 (189) | 25.2 (139) |
| A little worried | 35.4 (104) | 38.2 (208) | 41.2 (357) | 38.6 (389) | 38.6 (216) |
| Moderately worried | 13.4 (40) | 14.6 (82) | 19.5 (173) | 24.9 (264) | 23.8 (127) |
| Very worried | 5.2 (12) | 7.1 (46) | 13 (110) | 14.1 (134) | 9.2 (53) |
| Don't know | 6.5 (19) | 5 (25) | 4.5 (38) | 2.9 (32) | 3.1 (20) |
|  |  |  |  |  |  |
| **US** |  |  |  |  |  |
| Not at all worried | 39 (178) | 32.2 (200) | 22.8 (198) | 21.7 (233) | 23.4 (179) |
| A little worried | 35 (159) | 36.3 (225) | 39.9 (351) | 41 (443) | 42.5 (360) |
| Moderately worried | 15.7 (73) | 19.2 (120) | 21.1 (188) | 22 (258) | 19.6 (174) |
| Very worried | 6 (28) | 8.2 (55) | 11.2 (90) | 11.9 (126) | 10.5 (100) |
| Don't know | 4.3 (19) | 4 (20) | 5.1 (43) | 3.4 (42) | 4 (38) |

**Table S6. Adjusted logistic regression models predicting** **exposure to mostly negative news stories and perceptions of vaping harms from survey wave, country, and demographic covariates.**

|  | **Full sample (n=63,380)** | | | | | | | | | | | | | | | | | | | |  | | **Past 30-day vapers (n=9,442)** | | | | | |
| --- | --- | --- | --- | --- | --- | --- | --- | --- | --- | --- | --- | --- | --- | --- | --- | --- | --- | --- | --- | --- | --- | --- | --- | --- | --- | --- | --- | --- |
|  |  | **Exposure to mostly negative news stories about vaping** | | | | |  | **Accurate perception that vaping is less harmful than smoking** | | | |  | | | **Perception that vaping takes less than a year to harm users’ health** | | | | | |  | | **Worry that vaping will damage your health in the future** | | | | | |
|  | **n** | **%** | | **AOR (95% CI)** | **p** | |  | **%** | **AOR (95% CI)** | **p** | | |  | | | **%** | | **AOR (95% CI)** | | **p** | |  | **n** | **%** | | **AOR (95% CI)** | **p** | |
| **SURVEY WAVE** | | |  |  |  |  | |  |  | |  | | |  | | |  | |  |  | |  |  |  |  | | |  |
| 2017 (Jul-Aug) | 12067 | 15.8 | | 1.00 |  | |  | 68.2 | 1.00 |  | | |  | | | 23.6 | | 1.00 | |  | |  | 1090 | 19.2 | | 1.00 |  | |
| 2018 (Aug-Sep) | 11713 | 24.1 | | **1.74 (1.62-1.87)** | **<.001** | |  | 65.1 | **0.88 (0.83-0.94)** | **<.001** | | |  | | | 28.1 | | **1.29 (1.20-1.37)** | | **<.001** | |  | 1493 | 24.7 | | **1.34 (1.08-1.65)** | **.008** | |
| 2019 (Aug-Sep) | 11549 | 43.1 | | **4.14 (3.85-4.44)** | **<.001** | |  | 54.1 | **0.55 (0.51-0.58)** | **<.001** | | |  | | | 34.1 | | **1.68 (1.58-1.80)** | | **<.001** | |  | 2220 | 30.6 | | **1.81 (1.48-2.20)** | **<.001** | |
| 2020 (Feb-Mar) | 13564 | 52.7 | | **6.16 (5.75-6.59)** | **<.001** | |  | 45.8 | **0.39 (0.36-0.41)** | **<.001** | | |  | | | 40.6 | | **2.22 (2.09-2.37)** | | **<.001** | |  | 2751 | 34.0 | | **2.14 (1.77-2.59)** | **<.001** | |
| 2020 (Aug) | 14487 | 37.5 | | **3.17 (2.96-3.39)** | **<.001** | |  | 47.4 | **0.42 (0.40-0.45)** | **<.001** | | |  | | | 39.8 | | **2.12 (2.00-2.26)** | | **<.001** | |  | 1888 | 30.2 | | **1.81 (1.48-2.21)** | **<.001** | |
| **COUNTRY** |  |  | |  |  | |  |  |  |  | | |  | | |  | |  | |  | |  |  |  | |  |  | |
| England | 19851 | 24.7 | | 1.00 |  | |  | 69.8 | 1.00 |  | | |  | | | 25.5 | | 1.00 | |  | |  | 2244 | 24.5 | | 1.00 |  | |
| Canada | 20396 | 36.1 | | **1.85 (1.76-1.95)** | **<.001** | |  | 54.0 | **0.51 (0.49-0.54)** | **<.001** | | |  | | | 34.2 | | **1.63 (1.55-1.71)** | | **<.001** | |  | 3298 | 31.1 | | **1.30 (1.13-1.49)** | **<.001** | |
| US | 23133 | 43.3 | | **2.41 (2.29-2.54)** | **<.001** | |  | 44.6 | **0.35 (0.33-0.36)** | **<.001** | | |  | | | 40.3 | | **1.97 (1.87-2.07)** | | **<.001** | |  | 3900 | 30.1 | | **1.31 (1.14-1.51)** | **<.001** | |
| **AGE GROUP** |  |  | |  |  | |  |  |  |  | | |  | | |  | |  | |  | |  |  |  | |  |  | |
| 16-17 | 27468 | 34.3 | | 1.00 |  | |  | 52.8 | 1.00 |  | | |  | | | 34.4 | | 1.00 | |  | |  | 3544 | 28.5 | | 1.00 |  | |
| 18-19 | 35912 | 35.9 | | **1.09 (1.05-1.13)** | **<.001** | |  | 58.1 | **1.27 (1.22-1.32)** | **<.001** | | |  | | | 33.0 | | **0.95 (0.91-0.99)** | | **.009** | |  | 5898 | 29.3 | | 1.00 (0.90-1.12) | .964 | |
| **GENDER** |  |  | |  |  | |  |  |  |  | | |  | | |  | |  | |  | |  |  |  | |  |  | |
| Male | 23183 | 33.3 | | 1.00 |  | |  | 55.0 | 1.00 |  | | |  | | | 32.1 | | 1.00 | |  | |  | 3638 | 28.5 | | 1.00 |  | |
| Female | 40197 | 37.2 | | **1.21 (1.16-1.26)** | **<.001** | |  | 56.1 | **1.05 (1.01-1.09)** | **.010** | | |  | | | 35.5 | | **1.17 (1.13-1.22)** | | **<.001** | |  | 5804 | 29.5 | | 1.01 (0.91-1.13) | .787 | |
| **RACE/ETHNICITY** | | |  |  |  |  | |  |  | |  | | |  | | |  | |  |  | |  |  |  |  | | |  |
| White only | 39333 | 35.6 | | 1.00 |  | |  | 56.9 | 1.00 |  | | |  | | | 35.2 | | 1.00 | |  | |  | 6477 | 27.7 | | 1.00 |  | |
| Any other race/ethnicity | 22917 | 34.9 | | **0.90 (0.86-0.94)** | **<.001** | |  | 53.1 | **0.89 (0.85-0.92)** | **<.001** | | |  | | | 30.5 | | **0.76 (0.73-0.79)** | | **<.001** | |  | 2828 | 32.2 | | **1.23 (1.09-1.37)** | **<.001** | |
| Don't know/refused | 1130 | 20.0 | | **0.46 (0.39-0.55)** | **<.001** | |  | 40.7 | **0.45 (0.39-0.52)** | **<.001** | | |  | | | 28.8 | | **0.78 (0.67-0.90)** | | **.001** | |  | 137 | 40.3 | | **1.77 (1.20-2.61)** | **.004** | |

All data except sample n are weighted.

|  | **Full sample (n=63,380)** | | | | | | | | | | | | | | | | | | | |  | | **Past 30-day vapers (n=9,442)** | | | | | |
| --- | --- | --- | --- | --- | --- | --- | --- | --- | --- | --- | --- | --- | --- | --- | --- | --- | --- | --- | --- | --- | --- | --- | --- | --- | --- | --- | --- | --- |
|  |  | **Exposure to mostly negative news stories about vaping** | | | | |  | **Accurate perception that vaping is less harmful than smoking** | | | |  | | | **Perception that vaping takes less than a year to harm users’ health** | | | | | |  | | **Worry that vaping will damage your health in the future** | | | | | |
|  | **n** | **%** | | **AOR (95% CI)** | **p** | |  | **%** | **AOR (95% CI)** | **p** | | |  | | | **%** | | **AOR (95% CI)** | | **p** | |  | **n** | **%** | | **AOR (95% CI)** | **p** | |
| **SURVEY WAVE** | | |  |  |  |  | |  |  | |  | | |  | | |  | |  |  | |  |  |  |  | | |  |
| 2017 (Jul-Aug) | 12067 | 15.8 | | **0.24 (0.23-0.26)** | **<.001** | |  | 68.2 | **1.83 (1.72-1.94)** | **<.001** | | |  | | | 23.6 | | **0.59 (0.56-0.63)** | | **<.001** | |  | 1090 | 19.2 | | **0.55 (0.45-0.67)** | **<.001** | |
| 2018 (Aug-Sep) | 11713 | 24.1 | | **0.42 (0.39-0.45)** | **<.001** | |  | 65.1 | **1.61 (1.51-1.71)** | **<.001** | | |  | | | 28.1 | | **0.76 (0.72-0.82)** | | **<.001** | |  | 1493 | 24.7 | | **0.74 (0.62-0.88)** | **.001** | |
| 2019 (Aug-Sep) | 11549 | 43.1 | | 1.00 |  | |  | 54.1 | 1.00 |  | | |  | | | 34.1 | | 1.00 | |  | |  | 2220 | 30.6 | | 1.00 |  | |
| 2020 (Feb-Mar) | 13564 | 52.7 | | **1.49 (1.40-1.58)** | **<.001** | |  | 45.8 | **0.70 (0.66-0.75)** | **<.001** | | |  | | | 40.6 | | **1.32 (1.24-1.41)** | | **<.001** | |  | 2751 | 34.0 | | **1.19 (1.03-1.37)** | **.021** | |
| 2020 (Aug) | 14487 | 37.5 | | **0.77 (0.72-0.81)** | **<.001** | |  | 47.4 | **0.78 (0.73-0.82)** | **<.001** | | |  | | | 39.8 | | **1.26 (1.19-1.34)** | | **<.001** | |  | 1888 | 30.2 | | 1.00 (0.86-1.17) | .977 | |
|  |  |  | |  |  | |  |  |  |  | | |  | | |  | |  | |  | |  |  |  | |  |  | |
| **COUNTRY** |  |  | |  |  | |  |  |  |  | | |  | | |  | |  | |  | |  |  |  | |  |  | |
| Canada | 20396 | 36.1 | | 1.00 |  | |  | 54.0 | 1.00 |  | | |  | | | 34.2 | | 1.00 | |  | |  | 3298 | 31.1 | | 1.00 |  | |
| England | 19851 | 24.7 | | **0.54 (0.51-0.57)** | **<.001** | |  | 69.8 | **1.94 (1.85-2.04)** | **<.001** | | |  | | | 25.5 | | **0.61 (0.58-0.65)** | | **<.001** | |  | 2244 | 24.5 | | **0.77 (0.67-0.88)** | **<.001** | |
| US | 23133 | 43.3 | | **1.30 (1.24-1.36)** | **<.001** | |  | 44.6 | **0.67 (0.64-0.71)** | **<.001** | | |  | | | 40.3 | | **1.21 (1.15-1.27)** | | **<.001** | |  | 3900 | 30.1 | | 1.01 (0.89-1.14) | .889 | |

**Table S7. Associations between exposure to mostly negative news stories and perceptions of vaping harms and survey wave (2017-2020) and country, with Aug-Sep 2019 and Canada as the reference categories, adjusting for demographic covariates.**

All data except sample n are weighted.

**Table S8. Subgroup analyses among never smokers only: Associations between exposure to mostly negative news stories and perceptions of vaping harms and survey wave and country, adjusting for demographic covariates. Interactions between survey wave and country are also reported.**

| **All never smokers (n=40,288)** | | | | | | | | | | | |  | | | **Never smokers who are also past 30-day vapers (n=518)** | | | | | |  |  |
| --- | --- | --- | --- | --- | --- | --- | --- | --- | --- | --- | --- | --- | --- | --- | --- | --- | --- | --- | --- | --- | --- | --- |
|  |  | **Exposure to mostly negative news stories about vaping** | | |  | **Accurate perception that vaping is less harmful than smoking** | | |  | **Perception that vaping takes less than a year to harm users’ health** | | |  | | **Worry that vaping will damage your health in the future** | | | | |  |  |  |
|  | **n** | **%** | **AOR (95% CI)** | **p** |  | **%** | **AOR (95% CI)** | **p** |  | **%** | **AOR (95% CI)** | **p** | |  | | **n** | **%** | **AOR (95% CI)** | **p** | | | |
| **SURVEY WAVE** | |  |  |  |  |  |  |  |  |  |  |  | |  | |  |  |  |  | | | |
| 2017 (Jul-Aug) | 7882 | 15.9 | **1.00** |  |  | 66.9 | 1.00 |  |  | 26.1 | **1.00** |  | |  | | 26 | 15.5 | 1.00 |  | | | |
| 2018 (Aug-Sep) | 7441 | 24.8 | **1.79 (1.63-1.95)** | **<.001** |  | 63.7 | **0.87 (0.81-0.94)** | **.001** |  | 30.4 | **1.27 (1.17-1.37)** | **<.001** | |  | | 53 | 18.3 | 1.17 (0.65-2.08) | .605 | | | |
| 2019 (Aug-Sep) | 7135 | 44.8 | **4.45 (4.07-4.85)** | **<.001** |  | 52.7 | **0.54 (0.50-0.58)** | **<.001** |  | 36.1 | **1.62 (1.50-1.76)** | **<.001** | |  | | 143 | 29.6 | **2.23 (1.32-3.76)** | **.003** | | | |
| 2020 (Feb-Mar) | 7930 | 54.1 | **6.39 (5.86-6.96)** | **<.001** |  | 43.0 | **0.37 (0.34-0.40)** | **<.001** |  | 43.4 | **2.17 (2.01-2.34)** | **<.001** | |  | | 177 | 33.4 | **2.59 (1.55-4.35)** | **<.001** | | | |
| 2020 (Aug) | 9900 | 38.7 | **3.29 (3.03-3.58)** | **<.001** |  | 45.7 | **0.43 (0.40-0.46)** | **<.001** |  | 42.8 | **2.09 (1.94-2.26)** | **<.001** | |  | | 119 | 28.6 | **2.04 (1.20-3.48)** | **.008** | | | |
|  |  |  |  |  |  |  |  |  |  |  |  |  | |  | |  |  |  |  | | | |
| **COUNTRY** |  |  |  |  |  |  |  |  |  |  |  |  | |  | |  |  |  |  | | | |
| England | 11345 | 25.2 | **1.00** |  |  | 70.6 | 1.00 |  |  | 26.5 | 1.00 |  | |  | | 50 | 21.1 | 1.00 |  | | | |
| Canada | 13372 | 36.4 | **1.81 (1.70-1.93)** | **<.001** |  | 53.8 | **0.49 (0.46-0.52)** | **<.001** |  | 36.0 | **1.70 (1.60-1.81)** | **<.001** | |  | | 204 | 26.7 | 1.25 (0.84-1.85) | .272 | | | |
| US | 15571 | 43.9 | **2.35 (2.21-2.51)** | **<.001** |  | 41.3 | **0.29 (0.27-0.31)** | **<.001** |  | 43.7 | **2.13 (2.00-2.26)** | **<.001** | |  | | 264 | 29.4 | 1.45 (0.98-2.15) | .064 | | | |
|  |  |  |  |  |  |  |  |  |  |  |  |  | |  | |  |  |  |  | | | |
| **SURVEY WAVE * country interaction^1^** | | |  |  |  |  |  |  |  |  |  |  | |  | |  |  |  |  | | | |
|  | | **F (8,40280)=19.87 <.001** | | |  | **F (8,40280)=4.22 <.001** | | |  | **F (8,40280)=4.22 <.001** | | | |  | | F (8,1869)=1.16 .320 | | | | | |  |

All data except sample n are weighted.

^1^ Interactions were added in a second step to the regression models.

| **All ever smokers (n=14,543)** | | | | | | | | | | | |  | | | **Ever smokers who are also past 30-day vapers (n=996)** | | | | | |  |
| --- | --- | --- | --- | --- | --- | --- | --- | --- | --- | --- | --- | --- | --- | --- | --- | --- | --- | --- | --- | --- | --- |
|  |  | **Exposure to mostly negative news stories about vaping** | | |  | **Accurate perception that vaping is less harmful than smoking** | | |  | **Perception that vaping takes less than a year to harm users’ health** | | |  | | **Worry that vaping will damage your health in the future** | | | | |  |  |
|  | **n** | **%** | **AOR (95% CI)** | **p** |  | **%** | **AOR (95% CI)** | **p** |  | **%** | **AOR (95% CI)** | **p** | |  | | **n** | **%** | **AOR (95% CI)** | **p** | | |
| **SURVEY WAVE** | |  |  |  |  |  |  |  |  |  |  |  | |  | |  |  |  |  | | |
| 2017 (Jul-Aug) | 2717 | 15.9 | **1.00** |  |  | 71.7 | 1.00 |  |  | 20.8 | **1.00** |  | |  | | 57 | 15.8 | 1.00 |  | | |
| 2018 (Aug-Sep) | 2607 | 24.3 | **1.72 (1.47-2.02)** | **<.001** |  | 71.2 | 1.00 (0.88-1.15) | .944 |  | 25.7 | **1.32 (1.15-1.52)** | **<.001** | |  | | 100 | 19.7 | 1.26 (0.85-1.87) | .241 | | |
| 2019 (Aug-Sep) | 2726 | 43.0 | **3.93 (3.38-4.55)** | **<.001** |  | 58.2 | **0.57 (0.50-0.65)** | **<.001** |  | 34.3 | **1.95 (1.70-2.25)** | **<.001** | |  | | 259 | 31.2 | **2.30 (1.62-3.26)** | **<.001** | | |
| 2020 (Feb-Mar) | 3445 | 53.6 | **6.17 (5.35-7.11)** | **<.001** |  | 51.1 | **0.42 (0.37-0.47)** | **<.001** |  | 39.0 | **2.42 (2.12-2.76)** | **<.001** | |  | | 368 | 34.9 | **2.75 (1.96-3.87)** | **<.001** | | |
| 2020 (Aug) | 3048 | 36.7 | **3.02 (2.61-3.51)** | **<.001** |  | 54.1 | **0.46 (0.41-0.52)** | **<.001** |  | 34.5 | **1.99 (1.74-2.28)** | **<.001** | |  | | 212 | 28.7 | **2.11 (1.47-3.02)** | **<.001** | | |
|  |  |  |  |  |  |  |  |  |  |  |  |  | |  | |  |  |  |  | | |
| **COUNTRY** |  |  |  |  |  |  |  |  |  |  |  |  | |  | |  |  |  |  | | |
| England | 5019 | 24.7 | **1.00** |  |  | 72.6 | **1.00** |  |  | 25.6 | **1.00** |  | |  | | 183 | 25.0 | 1.00 |  | | |
| Canada | 4523 | 37.3 | **1.90 (1.71-2.11)** | **<.001** |  | 55.9 | **0.49 (0.45-0.54)** | **<.001** |  | 32.6 | **1.46 (1.32-1.61)** | **<.001** | |  | | 387 | 31.4 | 1.26 (0.99-1.59) | .059 | | |
| US | 5001 | 45.0 | **2.52 (2.27-2.80)** | **<.001** |  | 52.9 | **0.43 (0.39-0.48)** | **<.001** |  | 35.9 | **1.61 (1.45-1.79)** | **<.001** | |  | | 426 | 27.8 | 1.09 (0.86-1.39) | .470 | | |
|  |  |  |  |  |  |  |  |  |  |  |  |  | |  | |  |  |  |  | | |
| **SURVEY WAVE * country interaction^1^** | | |  |  |  |  |  |  |  |  |  |  | |  | |  |  |  |  | | |
|  | | **F (8,14535)=4.89 <.001** | | |  | **F (8,14535)=2.61 .007** | | |  | F (8,14535)=1.82 .068 | | | |  | | F (8,3363)=1.18 .307 | | | | | |

**Table S9. Subgroup analyses among ever (but not past 30-day) smokers only: Associations between exposure to mostly negative news stories and perceptions of vaping harms and survey wave and country, adjusting for demographic covariates. Interactions between survey wave and country are also reported.**

All data except sample n are weighted.

^1^ Interactions were added in a second step to the regression models.

| **All past 30-day smokers (n=8,549)** | | | | | | | | | | | |  | | | **Past 30-day smokers who are also past 30-day vapers (n=1,298)** | | | | | |  |
| --- | --- | --- | --- | --- | --- | --- | --- | --- | --- | --- | --- | --- | --- | --- | --- | --- | --- | --- | --- | --- | --- |
|  |  | **Exposure to mostly negative news stories about vaping** | | |  | **Accurate perception that vaping is less harmful than smoking** | | |  | **Perception that vaping takes less than a year to harm users’ health** | | |  | | **Worry that vaping will damage your health in the future** | | | | |  |  |
|  | **n** | **%** | **AOR (95% CI)** | **p** |  | **%** | **AOR (95% CI)** | **p** |  | **%** | **AOR (95% CI)** | **p** | |  | | **n** | **%** | **AOR (95% CI)** | **p** | | |
| **SURVEY WAVE** | |  |  |  |  |  |  |  |  |  |  |  | |  | |  |  |  |  | | |
| 2017 (Jul-Aug) | 1468 | 15.0 | **1.00** |  |  | 69.0 | **1.00** |  |  | 15.6 | **1.00** |  | |  | | 125 | 22.2 | 1.00 |  | | |
| 2018 (Aug-Sep) | 1665 | 20.4 | **1.52 (1.23-1.88)** | **<.001** |  | 61.7 | **0.76 (0.64-0.90)** | **.001** |  | 20.4 | **1.42 (1.16-1.74)** | **.001** | |  | | 220 | 31.5 | **1.54 (1.16-2.05)** | **.003** | | |
| 2019 (Aug-Sep) | 1688 | 32.4 | **2.82 (2.32-3.44)** | **<.001** |  | 54.0 | **0.52 (0.44-0.61)** | **<.001** |  | 21.3 | **1.46 (1.20-1.79)** | **<.001** | |  | | 283 | 30.6 | **1.56 (1.18-2.05)** | **.002** | | |
| 2020 (Feb-Mar) | 2189 | 43.1 | **4.82 (3.99-5.83)** | **<.001** |  | 49.1 | **0.40 (0.34-0.46)** | **<.001** |  | 28.9 | **2.27 (1.88-2.73)** | **<.001** | |  | | 416 | 33.3 | **1.85 (1.43-2.41)** | **<.001** | | |
| 2020 (Aug) | 1539 | 30.3 | **2.63 (2.15-3.23)** | **<.001** |  | 44.3 | **0.34 (0.29-0.41)** | **<.001** |  | 30.1 | **2.40 (1.97-2.92)** | **<.001** | |  | | 254 | 32.7 | **1.76 (1.33-2.33)** | **<.001** | | |
|  |  |  |  |  |  |  |  |  |  |  |  |  | |  | |  |  |  |  | | |
| **COUNTRY** |  |  |  |  |  |  |  |  |  |  |  |  | |  | |  |  |  |  | | |
| England | 3487 | 22.7 | **1.00** |  |  | 62.7 | **1.00** |  |  | 21.8 | **1.00** |  | |  | | 326 | 24.8 | 1.00 |  | | |
| Canada | 2501 | 31.4 | **1.76 (1.54-2.03)** | **<.001** |  | 51.4 | **0.63 (0.56-0.71)** | **<.001** |  | 24.5 | **1.24 (1.07-1.42)** | **.003** | |  | | 450 | 33.8 | **1.48 (1.21-1.80)** | **<.001** | | |
| US | 2561 | 34.2 | **2.10 (1.82-2.42)** | **<.001** |  | 48.4 | **0.52 (0.46-0.59)** | **<.001** |  | 24.6 | **1.28 (1.10-1.48)** | **.001** | |  | | 522 | 33.4 | **1.57 (1.29-1.91)** | **<.001** | | |
|  |  |  |  |  |  |  |  |  |  |  |  |  | |  | |  |  |  |  | | |
| **SURVEY WAVE * country interaction^1^** | | |  |  |  |  |  |  |  |  |  |  | |  | |  |  |  |  | | |
|  | | F (8,8541)=1.71 .091 | | |  | F (8,8541)=1.90 .055 | | |  | F (8,8541)=1.59 .122 | | | |  | | F (8,4186)=1.85 .063 | | | | | |

**Table S10. Subgroup analyses among past 30-day smokers only: Associations between exposure to mostly negative news stories and perceptions of vaping harms and survey wave and country, adjusting for demographic covariates. Interactions between survey wave and country are also reported.**

All data except sample n are weighted.

^1^ Interactions were added in a second step to the regression models.

| **All never vapers (n=38,349)** | | | | | | | | | | | |  |
| --- | --- | --- | --- | --- | --- | --- | --- | --- | --- | --- | --- | --- |
|  |  | **Exposure to mostly negative news stories about vaping** | | |  | **Accurate perception that vaping is less harmful than smoking** | | |  | **Perception that vaping takes less than a year to harm users’ health** | | |
|  | **n** | **%** | **AOR (95% CI)** | **p** |  | **%** | **AOR (95% CI)** | **p** |  | **%** | **AOR (95% CI)** | **p** |
| **SURVEY WAVE** | |  |  |  |  |  |  |  |  |  |  |  |
| 2017 (Jul-Aug) | 8276 | 15.9 | **1.00** |  |  | 64.6 | **1.00** |  |  | 26.3 | **1.00** |  |
| 2018 (Aug-Sep) | 7638 | 24.3 | **1.75 (1.6-1.91)** | **<.001** |  | 60.6 | **0.84 (0.78-0.91)** | **<.001** |  | 31.0 | **1.29 (1.19-1.40)** | **<.001** |
| 2019 (Aug-Sep) | 6414 | 42.1 | **4.04 (3.7-4.42)** | **<.001** |  | 49.7 | **0.52 (0.48-0.56)** | **<.001** |  | 37.1 | **1.71 (1.58-1.86)** | **<.001** |
| 2020 (Feb-Mar) | 7043 | 51.4 | **5.87 (5.38-6.4)** | **<.001** |  | 39.5 | **0.34 (0.32-0.37)** | **<.001** |  | 43.9 | **2.25 (2.08-2.43)** | **<.001** |
| 2020 (Aug) | 8978 | 36.7 | **3.05 (2.8-3.32)** | **<.001** |  | 42.8 | **0.41 (0.38-0.44)** | **<.001** |  | 42.9 | **2.11 (1.96-2.28)** | **<.001** |
|  |  |  |  |  |  |  |  |  |  |  |  |  |
| **COUNTRY** |  |  |  |  |  |  |  |  |  |  |  |  |
| England | 12254 | 24.1 | **1.00** |  |  | 68.4 | **1.00** |  |  | 26.6 | **1.00** |  |
| Canada | 12159 | 33.8 | **1.76 (1.65-1.88)** | **<.001** |  | 49.9 | **0.45 (0.42-0.48)** | **<.001** |  | 36.3 | **1.77 (1.66-1.88)** | **<.001** |
| US | 13936 | 42.1 | **2.40 (2.24-2.56)** | **<.001** |  | 37.7 | **0.27 (0.26-0.29)** | **<.001** |  | 44.7 | **2.27 (2.13-2.41)** | **<.001** |
|  |  |  |  |  |  |  |  |  |  |  |  |  |
| **SURVEY WAVE * country interaction^1^** | | |  |  |  |  |  |  |  |  |  |  |
|  | | **F (8,38341)=16.19 <.001** | | |  | **F (8,38341)=6.81 <.001** | | |  | **F (8,38341)=3.68 <.001** | | |

**Table S11. Subgroup analyses among never vapers only: Associations between exposure to mostly negative news stories and perceptions of vaping harms and survey wave and country, adjusting for demographic covariates. Interactions between survey wave and country are also reported.**

All data except sample n are weighted.

^1^ Interactions were added in a second step to the regression models.

| **All ever vapers (n=15,589)** | | | | | | | | | | | |  |
| --- | --- | --- | --- | --- | --- | --- | --- | --- | --- | --- | --- | --- |
|  |  | **Exposure to mostly negative news stories about vaping** | | |  | **Accurate perception that vaping is less harmful than smoking** | | |  | **Perception that vaping takes less than a year to harm users’ health** | | |
|  | **n** | **%** | **AOR (95% CI)** | **p** |  | **%** | **AOR (95% CI)** | **p** |  | **%** | **AOR (95% CI)** | **p** |
| **SURVEY WAVE** | |  |  |  |  |  |  |  |  |  |  |  |
| 2017 (Jul-Aug) | 2701 | 16.3 | **1.00** |  |  | 75.7 | 1.00 |  |  | 19.9 | **1.00** |  |
| 2018 (Aug-Sep) | 2582 | 24.4 | **1.72 (1.47-2.01)** | **<.001** |  | 74.9 | 0.96 (0.83-1.11) | .604 |  | 24.2 | **1.31 (1.13-1.51)** | **<.001** |
| 2019 (Aug-Sep) | 2915 | 46.0 | **4.35 (3.76-5.03)** | **<.001** |  | 58.8 | **0.48 (0.42-0.55)** | **<.001** |  | 33.1 | **1.94 (1.68-2.23)** | **<.001** |
| 2020 (Feb-Mar) | 3770 | 54.9 | **6.44 (5.60-7.40)** | **<.001** |  | 50.6 | **0.33 (0.29-0.37)** | **<.001** |  | 41.2 | **2.80 (2.45-3.19)** | **<.001** |
| 2020 (Aug) | 3621 | 41.0 | **3.53 (3.07-4.07)** | **<.001** |  | 53.2 | **0.38 (0.33-0.43)** | **<.001** |  | 37.7 | **2.38 (2.09-2.72)** | **<.001** |
|  |  |  |  |  |  |  |  |  |  |  |  |  |
| **COUNTRY** |  |  |  |  |  |  |  |  |  |  |  |  |
| England | 5353 | 26.9 | **1.00** |  |  | 73.6 | **1.00** |  |  | 24.9 | **1.00** |  |
| Canada | 4939 | 40.8 | **1.99 (1.80-2.20)** | **<.001** |  | 58.4 | **0.51 (0.47-0.56)** | **<.001** |  | 32.9 | **1.56 (1.41-1.72)** | **<.001** |
| US | 5297 | 46.5 | **2.37 (2.14-2.62)** | **<.001** |  | 51.6 | **0.39 (0.36-0.43)** | **<.001** |  | 39.0 | **1.89 (1.71-2.10)** | **<.001** |
|  |  |  |  |  |  |  |  |  |  |  |  |  |
| **SURVEY WAVE * country interaction^1^** | | |  |  |  |  |  |  |  |  |  |  |
|  | | **F (8,15581)=7.95 <.001** | | |  | **F (8,15581)=3.60 <.001** | | |  | **F (8,15581)=2.80 .004** | | |

**Table S12. Subgroup analyses among ever (but not past 30-day) vapers only: Associations between exposure to mostly negative news stories and perceptions of vaping harms and survey wave and country, adjusting for demographic covariates. Interactions between survey wave and country are also reported.**

All data except sample n are weighted.

^1^ Interactions were added in a second step to the regression models.

| **All past 30-day vapers (n=9,442)** | | | | | | | | | | | |  |
| --- | --- | --- | --- | --- | --- | --- | --- | --- | --- | --- | --- | --- |
|  |  | **Exposure to mostly negative news stories about vaping** | | |  | **Accurate perception that vaping is less harmful than smoking** | | |  | **Perception that vaping takes less than a year to harm users’ health** | | |
|  | **n** | **%** | **AOR (95% CI)** | **p** |  | **%** | **AOR (95% CI)** | **p** |  | **%** | **AOR (95% CI)** | **p** |
| **SURVEY WAVE** | |  |  |  |  |  |  |  |  |  |  |  |
| 2017 (Jul-Aug) | 1090 | 13.9 | **1.00** |  |  | 77.4 | **1.00** |  |  | 12.6 | **1.00** |  |
| 2018 (Aug-Sep) | 1493 | 22.8 | **1.79 (1.41-2.27)** | **<.001** |  | 72.9 | 0.82 (0.67-1.01) | .061 |  | 19.5 | **1.66 (1.31-2.10)** | **<.001** |
| 2019 (Aug-Sep) | 2220 | 42.4 | **4.55 (3.66-5.66)** | **<.001** |  | 63.5 | **0.51 (0.42-0.62)** | **<.001** |  | 24.7 | **2.18 (1.75-2.71)** | **<.001** |
| 2020 (Feb-Mar) | 2751 | 53.6 | **7.28 (5.87-9.02)** | **<.001** |  | 59.1 | **0.42 (0.35-0.50)** | **<.001** |  | 29.1 | **2.75 (2.22-3.39)** | **<.001** |
| 2020 (Aug) | 1888 | 34.8 | **3.23 (2.58-4.04)** | **<.001** |  | 59.6 | **0.43 (0.35-0.52)** | **<.001** |  | 28.2 | **2.66 (2.14-3.31)** | **<.001** |
|  |  |  |  |  |  |  |  |  |  |  |  |  |
| **COUNTRY** |  |  |  |  |  |  |  |  |  |  |  |  |
| England | 2244 | 23.0 | **1.00** |  |  | 68.9 | **1.00** |  |  | 20.6 | 1.00 |  |
| Canada | 3298 | 38.7 | **2.16 (1.87-2.49)** | **<.001** |  | 65.1 | 0.90 (0.79-1.03) | .119 |  | 26.6 | **1.34 (1.16-1.56)** | **<.001** |
| US | 3900 | 43.9 | **2.75 (2.38-3.18)** | **<.001** |  | 62.2 | **0.73 (0.64-0.84)** | **<.001** |  | 24.5 | **1.20 (1.03-1.39)** | **.017** |
|  |  |  |  |  |  |  |  |  |  |  |  |  |
| **SURVEY WAVE * country interaction^1^** | | |  |  |  |  |  |  |  |  |  |  |
|  | | F (8,9434)=1.73 .086 | | |  | **F (8,9434)=2.22 .023** | | |  | F (8,9434)=0.42 .910 | | |

**Table S13. Subgroup analyses among past 30-day vapers only: Associations between exposure to mostly negative news stories and perceptions of vaping harms and survey wave and country, adjusting for demographic covariates. Interactions between survey wave and country are also reported.**

All data except sample n are weighted.

^1^ Interactions were added in a second step to the regression models.

| **Full sample (n=63,380)** | | | | | | | | | | | |  | | | **Past 30-day vapers (n=9,442)** | | | | | |  |
| --- | --- | --- | --- | --- | --- | --- | --- | --- | --- | --- | --- | --- | --- | --- | --- | --- | --- | --- | --- | --- | --- |
|  |  | **Exposure to mostly negative news stories about vaping** | | |  | **Accurate perception that vaping is less harmful than smoking** | | |  | **Perception that vaping takes less than a year to harm users’ health** | | |  | | **Worry that vaping will damage your health in the future** | | | | |  |  |
|  | **n** | **%** | **AOR (95% CI)** | **p** |  | **%** | **AOR (95% CI)** | **p** |  | **%** | **AOR (95% CI)** | **p** | |  | | **n** | **%** | **AOR (95% CI)** | **p** | | |
| **SURVEY WAVE** | |  |  |  |  |  |  |  |  |  |  |  | |  | |  |  |  |  | | |
| 2017 (Jul-Aug) | 12067 | 15.8 | 1.00 |  |  | 68.2 | 1.00 |  |  | 23.6 | 1.00 |  | |  | | 1090 | 19.2 | 1.00 |  | | |
| 2018 (Aug-Sep) | 11713 | 24.1 | **1.74 (1.61-1.87)** | **<.001** |  | 65.1 | **0.88 (0.83-0.94)** | **<.001** |  | 28.1 | **1.29 (1.20-1.37)** | **<.001** | |  | | 1493 | 24.7 | **1.34 (1.08-1.65)** | **.008** | | |
| 2019 (Aug) | 8629 | 40.6 | **3.75 (3.48-4.05)** | **<.001** |  | 54.2 | **0.54 (0.51-0.58)** | **<.001** |  | 33.8 | **1.66 (1.55-1.78)** | **<.001** | |  | | 1610 | 30.1 | **1.78 (1.45-2.19)** | **<.001** | | |
| 2019 (Sep) | 2920 | 50.0 | **5.44 (4.92-6.02)** | **<.001** |  | 54.0 | **0.56 (0.51-0.62)** | **<.001** |  | 35.2 | **1.75 (1.58-1.93)** | **<.001** | |  | | 610 | 31.9 | **1.87 (1.44-2.43)** | **<.001** | | |
| 2020 (Feb/Mar) | 13564 | 52.7 | **6.15 (5.74-6.59)** | **<.001** |  | 45.8 | **0.39 (0.36-0.41)** | **<.001** |  | 40.6 | **2.22 (2.09-2.37)** | **<.001** | |  | | 2751 | 34.0 | **2.14 (1.77-2.59)** | **<.001** | | |
| 2020 (Aug) | 14487 | 37.5 | **3.17 (2.96-3.39)** | **<.001** |  | 47.4 | **0.42 (0.40-0.45)** | **<.001** |  | 39.8 | **2.12 (2.00-2.26)** | **<.001** | |  | | 1888 | 30.2 | **1.81 (1.48-2.21)** | **<.001** | | |
|  |  |  |  |  |  |  |  |  |  |  |  |  | |  | |  |  |  |  | | |
| 2019 (Aug)^2^ | 8629 | 40.6 | 1.00 |  |  | 54.2 | 1.00 |  |  | 33.8 | 1.00 |  | |  | | 1610 | 30.1 | 1.00 |  | | |
| 2019 (Sep)^2^ | 2920 | 50.0 | **1.45 (1.31-1.60)** | **<.001** |  | 54.0 | 1.03 (0.93-1.14) | .569 |  | 35.2 | 1.05 (0.95-1.17) | .355 | |  | | 610 | 31.9 | 1.05 (0.83-1.33) | .683 | | |
|  |  |  |  |  |  |  |  |  |  |  |  |  | |  | |  |  |  |  | | |
| **COUNTRY** |  |  |  |  |  |  |  |  |  |  |  |  | |  | |  |  |  |  | | |
| England | 19851 | 24.7 | 1.00 |  |  | 69.8 | 1.00 |  |  | 25.5 | 1.00 |  | |  | | 2244 | 24.5 | 1.00 |  | | |
| Canada | 20396 | 36.1 | **1.82 (1.73-1.92)** | **<.001** |  | 54.0 | **0.51 (0.49-0.54)** | **<.001** |  | 34.2 | **1.62 (1.55-1.71)** | **<.001** | |  | | 3298 | 31.1 | **1.30 (1.13-1.49)** | **<.001** | | |
| US | 23133 | 43.3 | **2.41 (2.29-2.54)** | **<.001** |  | 44.6 | **0.35 (0.33-0.36)** | **<.001** |  | 40.3 | **1.97 (1.87-2.07)** | **<.001** | |  | | 3900 | 30.1 | **1.31 (1.14-1.51)** | **<.001** | | |
|  |  |  |  |  |  |  |  |  |  |  |  |  | |  | |  |  |  |  | | |
| **SURVEY WAVE * country interaction^1^** | | |  |  |  |  |  |  |  |  |  |  | |  | |  |  |  |  | | |
|  | | **F (10,63370)=21.05 <.001** | | |  | **F (10,63370)=5.03 <.001** | | |  | **F (10,63370)=3.72 <.001** | | | |  | | F (10,9432)=1.68 .080 | | | | | |

**Table S14. Associations between exposure to mostly negative news stories and perceptions of vaping harms and survey wave (split into August vs. September 2019) and country, adjusting for demographic covariates. Interactions between survey wave and country are also reported.**

All data except sample n are weighted.

^1^ Interactions were added in a second step to the regression models.

^2^ Associations between September 2019 vs. August 2019 when adjusting the reference category to be August 2019.
